# Healthcare Access Challenges and Facilitators for Back Pain Across the Rural-Urban Continuum in Saskatchewan, Canada: Cross-Sectional Results From a Provincial-Wide Telephone Survey

**DOI:** 10.1177/11786329231193794

**Published:** 2023-08-25

**Authors:** Katie Crockett, Stacey Lovo, Alison Irvine, Catherine Trask, Sarah Oosman, Veronica McKinney, Terrence McDonald, Nazmi Sari, Bertha Carnegie, Marie Custer, Stacey McIntosh, Brenna Bath

**Affiliations:** 1 School of Rehabilitation Science, University of Saskatchewan, Saskatoon, SK, Canada; 2 Department of Biomedical Engineering and Health Systems, School of Engineering Sciences in Chemistry, Biotechnology, & Health, Royal Institute of Technology, Stockholm, Sweden; 3 Canadian Centre for Rural and Agricultural Health, University of Saskatchewan, Saskatoon, SK, Canada; 4 College of Medicine, University of Saskatchewan, Saskatoon, SK, Canada; 5 Departments of Family Medicine and Community Health Sciences, University of Calgary, Calgary, AB, Canada; 6 Department of Economics, University of Saskatchewan, Saskatoon, SK, Canada; 7 Patient Partner, University of Saskatchewan, Saskatoon, SK, Canada

**Keywords:** Low back pain, rural health, rehabilitation, health services, Indigenous

## Abstract

**Background:**

Chronic back pain is a common musculoskeletal disorder, disproportionately affecting rural and Indigenous people. Saskatchewan has a relatively high proportion of rural and Indigenous residents; therefore, understanding barriers and facilitators to accessing healthcare are needed to improve healthcare service delivery.

**Methods:**

A provincial-wide telephone survey explored experiences and perceived healthcare access barriers and facilitators among 384 Saskatchewan residents who experienced chronic low back pain. Chi-squared tests were performed to determine if people who lived in urban versus rural areas differed in the proportion who had accessed services from various healthcare practitioners. *T*-test and Mann-Whitney *U* analyses were conducted to determine differences between urban and rural, and Indigenous and non-Indigenous respondents.

**Results:**

Of 384 residents surveyed, 234 (60.9%) reported living in a rural location; 21 (5.5%) identified as Indigenous. Wait times (47%), cost (40%), travel (39%), and not knowing how to seek help (37%) were the most common barriers for Saskatchewan residents seeking care, with travel being the only barrier that was significantly different between rural and urban respondents (*P* ⩽ .001). Not knowing where to go to access care or what would help their low back pain (*P* = .03), lack of cultural sensitivity (*P* = .007), and comfort discussing problems with health care professionals (*P* = .26) were greater barriers for Indigenous than non-Indigenous participants. Top facilitators (>50% of respondents) included publicly funded healthcare, locally accessible healthcare services, and having supportive healthcare providers who facilitate referral to appropriate care, with urban respondents considering the latter 2 as greater facilitators than rural respondents. Telehealth or virtual care (*P* = .013) and having healthcare options nearby in their community (*P* = .045) were greater facilitators among Indigenous participants compared to non-Indigenous respondents.

**Conclusions:**

Rural, urban, Indigenous, and non-Indigenous people report overlapping and unique barriers and facilitators to accessing care for chronic low back pain. Understanding perceived access experiences will assist in developing more effective care models for specific communities or regions.

## Background

Chronic musculoskeletal pain is a leading cause of disability world-wide, with low back pain having the greatest disability burden.^1^ With disability burden (Disability burden is defined by the World Health Organization using the disability-adjusted life year [DALY], a time-based measure factoring in years of life lost due to premature mortality [YLLs] and years of life lost due to time lived in states of less than full health, or years of healthy life lost due to disability [YLDs].) predicted to grow exponentially in the next 2 decades, this places unsustainable strain on already stressed health systems.^1^ In Canada, there are growing concerns that the health system is not as responsive, accessible, and/or safe for everyone, and this is exacerbated in some geographic regions, such as rural and remote areas.^2^

Chronic back pain can negatively impact both individuals and the broader health system due to the resource intense care needs for patients. For example, it has been shown that patients who experience chronic low back have high rates of primary physician care visits,^3^ specialist consultations, diagnostic procedures,^4^ and opioid use,^5^ among others. Improving access to non-pharmaceutical back pain treatment options, like physiotherapy care, is an especially important public health issue in Canada in the midst of the current opioid crisis, given that early access to physiotherapy care among people with back pain can lead to substantially reduced (up to 89%) likelihood of opioid use.^6^

Despite emerging evidence highlighting reduced access to appropriate care among Canadians with chronic back pain,^7,8^ there is a sparsity of research focused specifically on rural, remote, and/or Indigenous populations in the Canadian setting.^9,10^ In Saskatchewan, 32% of the population lives in rural and remote areas^11^ and 17% (187 885) of Saskatchewan residents are Indigenous.^12^ Saskatchewan falls within the Métis Nation Homeland, where numbered treaties are encompassed by the land of the province. Lower numbers of practitioners specialized in the management of musculoskeletal pain, such as physical therapists (PTs), are available in rural areas.^13,14^ Both rural, remote, and/or Indigenous populations are approximately 30% more likely to have chronic musculoskeletal issues such as chronic back pain.^15^ There are complex historical, psychosocial, cultural, and environmental factors that influence general health and wellbeing among Indigenous (Various definitions of Indigenous appear in research literature. Within the scope of this paper, “Indigenous” refers to unique and different population groups including First Nations and Métis peoples, acknowledging the inherent limitations of this definition.) people, which are intertwined with barriers and facilitators to accessing healthcare.^16^

There are multiple clinical guidelines providing similar recommendations for managing low back pain; however, a substantial gap persists between evidence and practice.^17^ Current recommendations for opioids for chronic non-cancer pain^18^ and low back pain in Canada^19^ include referral to multidisciplinary or non-physician care providers such as physiotherapists or chiropractors. However, given documented access barriers to these services and other issues related to feasibility of implementing guidelines, they become challenging to follow in many clinical contexts.^20^

Although potential barriers to healthcare access among Canadians with chronic back pain have been identified through population-based secondary data analysis^7^ and through qualitative exploration of focused population groups (eg, farmers,^21^ spinal triage service,^22^), no study has evaluated both barriers and facilitators of access to care between rural and urban residents in Saskatchewan, as well as Indigenous and non-Indigenous people. Having a better understanding of the barriers and facilitators that are unique to these populations may assist in developing more successful care models for people living with chronic low back pain. This study aimed to determine the perceived healthcare access barriers and facilitators among people who live with chronic back pain in Saskatchewan, and determine if differences exist among urban and rural residents, as well as Indigenous people.

## Methods

This was a cross-sectional population-based phone survey, part of a multi-phase study with detailed methodology previously published.^23^ Participants included Saskatchewan residents, 18 years of age or older, who had experienced lower back pain (defined as pain localized between the bottom of the rib cage and the bottom of the buttocks, that may or may not include leg pain) for at least 3 months. The Canadian Hub for Applied and Social Research (CHASR), University of Saskatchewan-based research support and consulting service, conducted a random probability telephone survey from May 5th to May 25th, 2022. An estimated sample of 383 was based on: the estimate that 20% of the adult population has chronic back pain^15^; the adult population of Saskatchewan according to most recent Census data^24^ (843 975); and a margin of error of ±5.00%. Oversampling of rural areas was performed in order to reflect the higher prevalence of CBP.^15,25^ VoxcoCATI software was used to dial telephone numbers at random. Telephone numbers were called up to 5 times without a response before the number was discarded. Participants were asked demographic questions, as well as experiences in health care access and use for their chronic low back pain. Findings from concurrent qualitative research^23^ examining barriers and facilitators to healthcare in Saskatchewan for chronic low back pain informed the development of our population-based phone survey questions focused on exploring perceived health care access barriers and facilitators among rural, remote, and urban patients with chronic back pain, with the opportunity for participants to report Indigenous status (Appendix 1). A comprehensive review describing the complex factors influencing general health and wellbeing among Indigenous people is beyond the scope of this manuscript; however, our commitment to understanding the unique needs of Indigenous people in Saskatchewan to further a respectful partnership with and among Indigenous community members is aligned with, and informed by, the United Nations Declaration on the Rights of Indigenous Peoples (UNDRIP)^26^ and the TRC Calls to Action.^27^ Our research approach is further described in our overarching protocol.^23^

The phone survey questions included both closed and open-ended questions. Descriptive analysis was conducted for demographic variables, as well as duration of back pain and health care services accessed for back pain. Chi-squared tests were performed to determine if people who lived in urban versus rural areas differed in the proportion who had accessed services from the listed health care practitioners. Responses to barriers were on a 4-point unimodal and symmetrical interval ranging from not at all a barrier, a minor barrier, a moderate barrier, to a serious barrier. Responses to facilitators were on a 4-point interval ranging from not at all a help, a minor help, a moderate help, or a major help. Given that data were normally distributed and with equal variances for majority of the variables, *T*-test analyses were conducted on all normally distributed variables with equal variances to determine if urban and rural respondents differed in the extent to which they faced barriers in getting the care they need for their back pain, and if there were any differences in facilitating factors in accessing care for their back pain.^28^ For the variables that were not normally distributed, Mann-Whitney *U* tests were conducted. The same methodology was used to determine if Indigenous and non-Indigenous respondents differed in the extent to which they faced barriers or facilitators in getting the care they need for their back pain. Although formal qualitative analysis was not completed, participant quotations were collected to highlight key findings from quantitative analyses.

Ethical approval for this research was provided by the University of Saskatchewan Behavioural Research Ethics Board (Beh REB #1973).

## Results

The survey generated a response rate of 12.6% and resulted in 384 completed interviews among randomly-selected Saskatchewan residents who have been experiencing low back pain for at least 3 months (ie, over 3000 people were contacted, with 384 people choosing to participate). Descriptive characteristics are reported in Table 1. One individual did not disclose rural vs urban residence, so was removed from analyses.

**Table 1.** Demographic characteristics.

Demographic characteristic	Total (n = 384) % of total (n)	Urban (n = 149) % of urban (n)	Rural (n = 234) % of rural (n)
Gender			
Man	33.9 (130)	36.2 (54)	32.5(76)
Woman	65.4 (251)	63.8 (95)	66.2 (155)
Other*	0.8 (3)	0 (0)	1.3(3)
Age			
20-29	1.0 (4)	0.7 (1)	0.9 (2)
30-39	1.3 (5)	0.7 (1)	1.7 (4)
40-49	4.2 (16)	5.4 (8)	3.4 (8)
50-59	12.8 (49)	16.1 (24)	10.7 (25)
60-69	29.0 (111)	23.5 (35)	32.5 (76)
70-79	32.9 (126)	32.2 (48)	33.3 (78)
80-89	14.9 (57)	16.8 (25)	13.7 (32)
90-99	2.1 (8)	2.7 (4)	1.7 (4)
Education			
Less than grade 12	19.1 (73)	12.1 (18)	23.5 (55)
Completed high school	21.4 (82)	18.8 (28)	23.1 (54)
Trade/technical school/college	27.2 (104)	21.5 (32)	30.8 (72)
Some university	10.2 (39)	12.8 (19)	8.5 (20)
Bachelor’s degree	16.4 (63)	26.2 (39)	10.3 (24)
Graduate degree	4.7 (18)	8.1 (12)	2.1 (5)
Employment status			
Work full-time	16.9 (65)	15.4 (23)	17.5 (41)
Work part-time	12.3 (47)	11.4 (17)	12.8 (30)
Caring for a child/family member	1.6 (6)	0.7 (1)	2.1 (5)
Unemployed	2.3 (9)	4.0 (6)	1.3 (3)
Disabled, unable to work	6.5 (25)	6.7 (10)	6.4 (15)
Student	0.8 (3)	0.7 (1)	0.9 (2)
Retired	56.5 (217)	59.7 (89)	54.7 (128)
Annual household income ($CAD)			
Less than $15 000	6.5 (25)	6.7 (10)	6.4 (15)
$15 000 to less than $30 000	14.1 (54)	11.4 (17)	15.8 (37)
$30 000 to less than $60 000	19.3 (74)	22.1 (33)	17.5 (41)
$60 000 to less than $100 000	16.7 (64)	20.1 (30)	14.1 (33)
$100 000 or more	15.1 (58)	14.8 (22)	15.4 (36)
Indigenous**			
Indigenous	5.5 (21)	4 (6)	6.4 (15)
Non-Indigenous	94.0 (361)	96 (143)	92.7 (217)

* An option for gender identity was provided. Three participants selected “other” but did not specify their gender identify beyond this.

** Within the scope of this paper, “Indigenous” refers to unique and different population groups including First Nations and Métis peoples.

**Figure 1.**
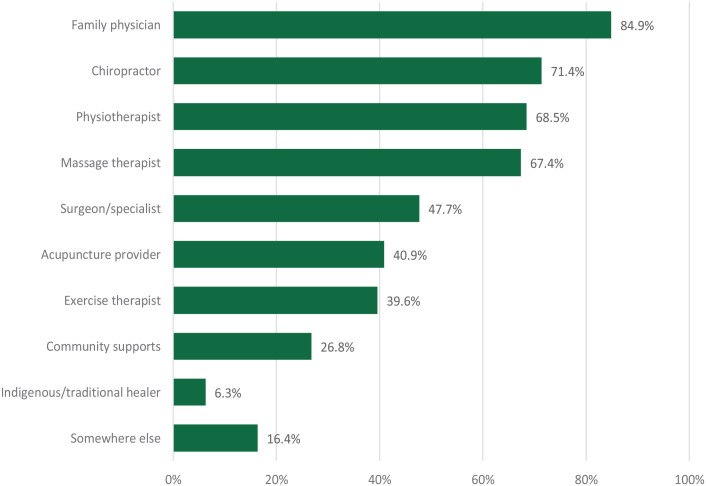
Healthcare/support services accessed for low back pain.

Health care practitioners from whom participants received care for their low back pain are reported in Figure 1. Seeing a family physician for their back pain was most common (84.9%), followed by a chiropractor (71.4%), physiotherapist (68.5%), and massage therapist (67.4%). More than half of respondents had accessed services from at least one of those 4 health care practitioners. Almost all the respondents (89.6%; n = 344) had taken over the counter medication (eg, Tylenol, Advil) and more than half (58.6%; n = 225) had taken medication prescribed by a health care provider.

Chi-squared tests revealed that significantly more urban respondents had accessed care for their back pain from family physicians, physiotherapists, and community supports (Table 2).

**Table 2.** Differences in healthcare/support services accessed between rural and urban respondents.

Health practitioner/service	*X* ^2^	Urban respondents who have accessed service (%)	Rural respondents who have accessed service (%)
Family Physician	χ^2^(1) = 4.36, *P* = .037	90.0	82.1
Physiotherapist	χ^2^(1) = 5.14, *P* = .023	76.4	65.4
Community Supports	χ^2^(1) = 9.00, *P* = .003	35.8	21.7

For health practitioners from whom respondents had *not* received care, respondents were asked to indicate why not. Suggestions of possible reasons included cost, wait time, travel time/location, a lack of interest, or they could provide their own response that did not fall into one of those categories. A lack of interest was the most common reason for not accessing services from all listed health care practitioners, with the exception of surgeons/specialists where not receiving a referral was the most common barrier (12.8%, n = 53), followed by the belief that their back pain was not bad enough (7.4%; n = 28) (Percentages reported are a proportion of all respondents.). Not having a family physician was the most common “other” reason for not accessing a family physician (3.4%, n = 13), with this being a greater barrier for rural respondents (rural: 4.7%, n = 11; urban: 1.3%, n = 2). Cost was a notable barrier for receiving services from physiotherapists (5.5%; n = 21), massage therapists (5.5%; n = 21), community supports (4.7%; n = 18), exercise therapists (4.4%; n = 17), acupuncture providers (4.2%; n = 16), and chiropractors (2.6%; n = 10). Not having access to care services provided by a particular healthcare practitioner/service also was a significant barrier in relation to community supports (21.6%; n = 83), exercise therapists (10.4%; n = 40), Indigenous or traditional healers (10.4%; n = 40), and acupuncture providers (8.1%; n = 31). Lack of awareness about the care provided by a health care practitioner impeded access to Indigenous and traditional healers (24.0%; n = 92) and exercise therapists (12.5%; n = 48).

### Barriers

**Figure 2.**
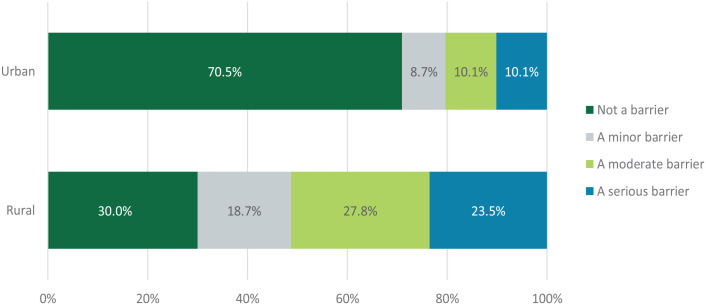
Differences in travel as a barrier to access healthcare between urban and rural respondence.

We investigated whether barriers to care were similar in both urban and rural participants. Rural respondents reported travel to access health services, *t*(338) = −7.51, *P* < .001 (urban: *M* = 1.59, SD = 1.03; rural: *M* = 2.45, SD = 1.15) to be a greater barrier than urban respondents (Figure 2).

*T*-test analyses revealed that Indigenous respondents report not knowing where to go or what will help, *t*(373) = 1.91, *P* = .03 (Indigenous: *M* = 2.60, SD = 1.14; non-Indigenous: *M* = 2.09, SD = 1.17) to be a greater barrier than non-Indigenous respondents.

**Figure 3.**
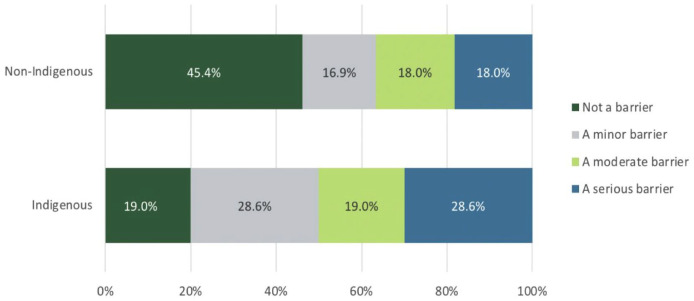
Differences in “Not knowing where to go” as a barrier to access healthcare between Indigenous and non-Indigenous respondents.

Mann-Whitney *U* analyses indicated that Indigenous respondents report cultural sensitivity among practitioners, *U* = 1689; *P* = .007 (Indigenous: Mdn = 1, SE = 0.21; non-Indigenous: Mdn = 1.0, SE = 0.035) as well as comfort discussing problems with health care professionals, *U* = 2951; *P* = .026 (Indigenous: Mdn = 1, SE = 0.22; non-Indigenous: Mdn = 1.0, SE = 0.04) to be greater barriers than non-Indigenous respondents (Figure 3).

### Facilitators

A *t-*test analysis of urban and rural differences on facilitators to receiving care for back pain revealed that urban respondents consider health care nearby in their community, *t*(371) = 3.06, *P* = .001 (urban: *M* = 2.90, SD = 1.22; rural: *M* = 2.51, SD = 1.19) and health care providers who help get care, *t*(368) = 2.14, *P* = .017 (urban: *M* = 2.99, SD = 1.14; rural: *M* = 2.73, SD = 1.09) as greater facilitators than rural respondents.

A *t-*test analysis of Indigenous and non-Indigenous differences on facilitators to receiving care for back pain revealed that Indigenous respondents consider telehealth or virtual care *t*(355) = 2.70, *P* = .004 (Indigenous: *M* = 2.63, SD = 1.26; non-Indigenous: *M* = 1.92, SD = 1.11) and having health nearby in their community, *t*(370) = 1.63, *P* = .05 (Indigenous: *M* = 3.11, SD = 1.10; non-Indigenous: *M* = 2.64, SD = 1.21) to be greater facilitators than non-Indigenous respondents.

The survey items were predominantly quantitative with some open-ended questions. Formal qualitative analysis did not take place. However, quotes highlighted key findings (Table 3).

**Table 3.** Barriers and facilitators to access care (qualitative responses).

Barrier	Facilitator
Perceived (lack of) access to services based on location	
*It’s just getting somebody to take me in. To travel to get there because I don’t drive. I recently lost my family physician. I don’t know where to go to.* (rural, non-Indigenous)	*The fact that I live in a city. I have easy access.* (urban, non-Indigenous)
*When your back is really sore, getting in the car and traveling is very difficult.* (rural, non-Indigenous)	*The fact that I’m close to everything is the best.* (urban, non-Indigenous)
Perceived (lack of) quality of health care services	
*I find the healthcare system don’t give a shit. You talk to some about it and they just write it down on paper but they don’t do anything about it*. (urban, non-Indigenous)	*I found that the professionals I have dealt with over the years are very committed people. They do the utmost to make things easier for you.* (urban, non-Indigenous)
*Because I’m 82 and nobody wants to do anything, so it’s “sweep it under the rug, you’re going to die next year.”* (urban, non-Indigenous)	*Consistent care by the same people because they know you and they believe you when you say you have pain.* (urban, Indigenous)
*I go to the doctor and tell them this hurts, this hurts, this hurts. All the doctors do is give you pills. Give you pills and that’s it.* (rural, Indigenous)	
Perceived (lack of) knowledge/awareness	
*Who does one go to? What professional health care provider do I approach?* (urban, non-Indigenous)	*Probably my own personal knowledge plus I read a lot, research a lot, listen to talks when they are talking about back pain.* (urban, non-Indigenous)
*Lack of knowledge of what services are going to be effective. It’s like an open sea of random things.* (urban, non-Indigenous)	*Maybe just the availability of information on the internet.* (rural, non-Indigenous)
*I didn’t think they can do anything about it.* (urban, non-Indigenous)	*Being a [health care provider] sure helps.* (urban, non-Indigenous)
Wait times	Navigating healthcare
*Wait lists are through the roof, and since I have such a chronic back pain, it’s not considered a priority, so it makes it longer, and if you’re not bad enough they do not [prioritize you].* (urban, non-Indigenous)	*My family doctor is very conscientious of trying to get me to people.* (rural, non-Indigenous)
*Like I said, it is hard and we don’t have a lot of physiotherapists and the ones we have are good but takes a long time to access them*. (rural, non-Indigenous)	*I have a physiotherapist who specializes in back pain and she helped me in get in touch with other back pain specialists.* (urban, non-Indigenous)
Costs	Family support
*Mostly cost and couldn’t get a physiotherapist without insurance and ran out of money and savings. . .* (urban, non-Indigenous)	*My daughter helps me sometimes to get to where I need to go.* (rural, Indigenous)
*It is hard to find someone that is covered by the medical system.* (urban, non-Indigenous)	*Family being able to drive me.* (urban, non-Indigenous)
Other responsibilities	Self-management strategies
*I know I should be going to a gym. But I can’t leave my husband.* (urban, non-Indigenous)	*Just doing it on my own. . .whatever I can do.* (rural, non-Indigenous)
*The only thing that is a major problem is working and trying to find time without taking time off work, even just making a doctor’s appointment without having to take time off.* (urban, non-Indigenous)	*If my back hurts, I go to my chiropractor, he gives me an exercise and do some of them until it goes away. I might do it once or twice a day to help until it’s gone.* (rural, non-Indigenous)
	*I don’t want to drive 30* *kms. I do yoga online.* (rural, non-Indigenous)
COVID-19 pandemic	
*First of all, you couldn’t get into the physician’s office. For a long time my GP wouldn’t come into the office. For a long time she would do it over the phone. How can you diagnose someone over the phone*? (rural, non-Indigenous)	*Healthcare plans waived the doctor referral requirement.* (urban, non-Indigenous)
*Simply because of doctors not wanting to actually see you. They just prefer a phone call and that’s fine for prescription when you can order things on the phone. But with my history with my back, it’s not something that you can diagnose over the phone* (rural, non-Indigenous)	*We have more access to the doctor by talking to them virtually.* (rural, non-Indigenous)
*Doctors do not want to see you, they want to talk to you on the phone and it is hard to diagnose when they can’t see the problem.* (rural, non-Indigenous)	*Now I can phone the doctor and he phones me back. I don’t have to go into town to access my doctor. that was a good thing for me* (rural, non-Indigenous)
*Just being able to make any in-person visit was extremely impossible, trying to explain to someone over the phone how it hurts is fairly difficult.* (urban, non-Indigenous)	
*Most doctors that I am aware of have had to scale down the patients that they can see because of social distancing. Some doctors refuse to do phone appointments and many don’t know how to do a zoom appointment*. (rural, non-Indigenous)	
*umm I don’t feel a phone consult does very good and I’m not wanting to go to ER, so I’m putting it off due to Covid.* (rural, non-Indigenous)	

## Discussion

A telephone survey of 384 Saskatchewan residents with chronic low back pain was completed in May 2022. Respondents indicated the type of health care services they had accessed, what barriers to access they have encountered, and what factors serve as facilitators to accessing care for their back pain. Many barriers and facilitators were common among urban and rural residents, but there were some differences. For example, rural respondents were less likely than urban respondents to have accessed care from family physicians, physiotherapists, and community supports. Rural respondents also encountered a greater barrier in relation to the need to travel to access health care services. Rural respondents were also less likely than urban respondents to perceive “nearby health services and providers who help them get care” as a facilitator to their care. Indigenous respondents reported a greater barrier to accessing care based on not knowing where to go or who to access for their chronic low back pain, cultural sensitivity, and comfort in discussing their problems with healthcare professionals. These findings are in line with prior research demonstrating increased likelihood of having chronic back pain among those living in rural or remote areas of Saskatchewan and Indigenous people,^25^ combined with reduced availability of musculoskeletal healthcare providers in rural and remote areas of Saskatchewan,^14^ and ongoing evidence of systemic racism existing in our healthcare system.^29^

For most Canadians with chronic back disorders, a family doctor is their primary point of contact within the health-care system, with a majority of Canadians only seeking care from a family doctor and not accessing care from a musculoskeletal care provider like a physiotherapist.^7^ Our result is consistent with this, as most participants have accessed a family physician (84.9%), with rural respondents less likely to have accessed a family physician, but still fairly high rate of access. Among older respondents (mean age of 68.6 years), with longstanding persistent pain (more than half of respondents experiencing low back pain for at least 10 years), it is not surprising that respondents will have had more experience in accessing various providers and during different iterations of the health care system. However, with more recent system changes, the Canadian Medical Association has acknowledged a “critical” family physician shortage, leaving 1 in 5 Canadians without a family physician.^30,31^ Contrary to 84.9% of participants stating that they have accessed a family physician, the most frequent barrier to accessing a family physician was that respondents did not have a family physician. This was more problematic with rural respondents and likely underreported in our data since it was not explicitly asked, but rather reported voluntarily as an “other” reason for not accessing a family physician. With a growing population that is increasing in age, and experiencing more chronic health concerns, it is becoming more difficult for family physicians to meet the diverse needs of patients.^32^ Given the Canadian primary care crisis,^33^ it is becoming more important to focus and invest in further development of team-based models of care^33^ or non-physician led models that have been shown to improve wait times and patient outcomes for low back pain care.^34-37^

There are few known interdisciplinary care models focusing on addressing the unique needs and context of rural or Indigenous communities in Saskatchewan and Canada.^10^ Many people with low back pain are still uncertain how to access non-pharmaceutical care without the guidance of health care providers like family physicians. Secondly, research has demonstrated inadequacies in our healthcare system including bias in pain assessment and treatment recommendations and consistent evidence of inequities based on colonization and systemic racism that can lead to avoidance of care.^29,38^ Consistent with prior research,^21^ wait times and costs emerged as the most common barriers among survey respondents with approximately 47% and 40%, respectively, respondents indicated that not knowing which services they should access or what will help was found as a significant barrier (37%) to accessing care for low back pain across urban, rural, remote, as well as Indigenous populations, with some Indigenous participants reporting that they did not feel healthcare providers believed their reports of pain. Indigenous respondents felt that the lack of cultural sensitivity among practitioners, and not feeling comfortable discussing their pain with their healthcare providers were barriers to accessing care. Although this research did not explicitly examine racism in healthcare, there is an important need to consider models of care and health care policies in ways that redress racism and mitigate harm for Indigenous peoples accessing care for low back pain.^29^ A main facilitator to accessing healthcare was having a healthcare provider who helps to get care (34.6% of respondents), providing appropriate referrals or educating on existing services that are appropriate at the right time. This is in line with current research in Saskatchewan where chronic pain patient expressed challenges with navigating the health system.^39^ Further research is needed on how best to educate the public and healthcare providers on anti-racism in healthcare, evidence based guidelines, and existing care options for chronic low back pain.^40,41^ Where appropriate, this could be without the need to first access a family physician or waiting to see a specialist to initiate care.^6^

Accessing a physiotherapist as a first point of contact has been shown to result in lower utilization of high-cost medical services, as well as lower opioid use.^6^ For many Saskatchewan residents, accessing a physiotherapist is not possible due to associated travel or cost barriers. A 2017 report from the CLHIA‒Canadian Life and Health Insurance Association^42^ estimates that 25 million Canadians have extended health care benefits. However, many patients in Canada still pay out of pocket for community-based, non-pharmacological services, such as those offered by physiotherapists, chiropractors, and psychologists when particular services are not covered or when certain benefits have been exhausted. Universal health-care coverage is typically limited to some prescription medications, diagnostic imaging, or referral to publicly funded medical specialists.^8^ In addition, remote Indigenous people have added jurisdictional challenges to the complexities to accessing care from musculoskeletal providers, as there is no funding for physiotherapy outside hospitals under Non-Insured Health Benefits (NIHB).^43^ All combined, this creates an issue of inequitable access, where not all patients can afford long-term treatment to manage chronic pain, particularly patients who are low-income or do not have private health insurance. These factors perpetuate inequities in low back pain experiences and outcomes among Indigenous peoples. When asked about facilitators to getting the care they need for their back pain, more than a third of respondents indicated that publicly funded health care (41.1%) was a major help.

Most respondents had accessed services from a range of different health care practitioners; most commonly, respondents accessed health care services from family physicians, chiropractors, physiotherapists, and massage therapists, with access rates ranging from 65% to 85%. Common reasons for not accessing certain health care services included a lack of interest in that service, not having received a referral, costs, not having nearby access, and a lack of knowledge or awareness of a particular service or health care provider, with the most common response being a lack of interest. This may be related to other associated barriers over the long-term, leading to “giving up” or may be related to a lack of awareness, as illustrated by these quotes from respondents: “*I don’t think they can do anything*”; “*They can’t do anything, it’s the bone, not much they can do*”; “*No solution at the moment*.” Other participant responses associated with being not interested in certain providers were related to already having accessed a different provider, relying on self-management strategies with majority describing self-guided coping strategies, or simply that they don’t think that specific practitioner would help. Some quotes: “*Saskatchewan doesn’t have access to healthcare like* that.” (referring to physiotherapy, which is available in Saskatchewan). These examples demonstrate an implied lack of awareness; however, majority of respondents indicated that they were not interested without providing any further explanation. This response requires further investigation into the complexity associated with not being interested, relative to other existing barriers, as well as current state of their chronic back pain and appropriateness of various services given individual circumstances.

The need to travel to access services also emerged as a substantial barrier overall (39%) and was a greater barrier for those living in rural areas of Saskatchewan. This is not surprising, given the limited distribution of physiotherapy services in rural and remote Saskatchewan and lower use of these services among people living in rural communities.^7,14,44^ On the other hand, having healthcare nearby that did not require extended travel time was a major facilitator for 34.9% of respondents. A hub and spoke model of care has been demonstrated to be an effective model of care for chronic pain, providing core services within communities, or at the spoke.^45^ Hub and spoke models are starting to integrate videoconferencing within the model of care, with ongoing efforts in establishing toolkits and other resources to enhance this model to care.^46^

Despite recent research demonstrating the value of virtual care in certain contexts, telehealth/virtual care rated lowest among facilitators with our respondents, with 48.2% of the sample indicating it was not at all a help. Virtual care has been shown to be an effective way of providing assessment and care for chronic low back pain^47-49^; unfortunately, many respondents perceived this question as a telephone call and majority of the respondents indicated that phone call appointments were occurring due to the COVID-19 pandemic. In Saskatchewan, Telehealth is defined as “linking patients to health care teams using highly secure videoconferencing technologies.”^50^ This survey was deployed during the COVID-19 pandemic, when phone consultations were becoming a predominant method of care provision with family physicians, with 58.2% of care being provided over the phone as of January 2021.^51^ It is also possible that some respondents did not have the opportunity to access telehealth or virtual care given the reported duration of symptoms and virtual care being an emerging platform for care provision^52^; therefore, this response may reflect a lack of availability of virtual care versus a negative experience with virtual care. The nature of the phone survey did not allow for clarification of this finding. This should be further explored with future research, and future research should clearly distinguish phone consultation versus videoconferencing. Despite these limitations to our study, Indigenous respondents reported that telehealth or virtual care was one of the main facilitators to accessing care, with almost 30% of all respondents indicating that it was a moderate-major help to accessing care. Virtual care allows Indigenous people living outside urban centers to remain in their community, where they can be surrounded by family, language, and important cultural supports through their chronic pain experience when they may feel the need for enhanced support from family members. Given the rapid uptake of virtual care in response to the COVID-19 pandemic, practitioners must now consider the format of care provision post-pandemic (ie, in-person, video, telephone) to ensure optimal patient outcomes.^53^ With virtual care as an emerging area of practice,^52^ further evidence is needed to establish quality improvement measures, appropriate training for practitioners including cultural responsiveness and anti-racism training and policies, as well as clinical guidelines in order to provide assurance for patients that they are receiving high quality, equitable care.

### Strengths and limitations

This population-based study had a number of notable strengths. First, there was strong sampling methodology applied, with community-informed questions integrating principles of patient-oriented research. The study captured some of the shifts in care related to the COVID-19 pandemic, which is realistic of current healthcare provision and capturing current experiences. However, there were several limitations to consider as well. The nature of the telephone survey relied of self-report and perceived contributors to accessing healthcare. Our response rate is likely an underestimate, as some respondents who refused to participate may not have qualified (ie, did not have back pain) had they progressed to the point in the survey where interviewers could ask them eligibility questions. Although we determined the sample size a priori based on the estimated prevalence of chronic back pain, the data analyses (eg, comparing rural vs urban and Indigenous vs non-Indigenous) may have a risk of type I errors, given the multiple tests included. To further elucidate these relationships, future research should be done with larger sample sizes. Since only 5.5% of the respondents were Indigenous (17% of the Saskatchewan population is Indigenous); the sample underrepresents the Indigenous population in the province. The nature of the phone survey may not have allowed time and space to build the trust and rapport necessary for individuals to share the true depth of their experiences, especially as they relate to systemic racism in healthcare. Further community-led research is needed to ensure Indigenous experiences are understood, so appropriate and relevant advocacy and care can be developed to enhance equitable access to culturally responsive services. In addition, the nature of the phone survey did not allow for discussion, clarification, or follow-up questions on responses to address assumptions or perceptions on the meaning of survey questions. For example, based on responses, it was clear that the question on telehealth/virtual care was influenced by the COVID-19 pandemic changes in provision on care, and many participants responded in a way that reflected phone consultation care as a response to the pandemic. Despite the limitations, future research can build on the knowledge gained in this phone survey to further explore these barriers and facilitators to accessing healthcare for Saskatchewan residents.

## Conclusion

Our findings suggest that rural Saskatchewan residents are not as easily able to get the same types of care for their back pain as urban respondents. They face greater barriers and have fewer facilitating factors. Our findings further suggest that Indigenous people have unique barriers to accessing care, including not knowing where to go or who could help with their low back pain and experiences with providers demonstrating lack of cultural sensitivity. Indigenous people may experience chronic back pain in diverse and unique ways that may be different from the general population.^16^ Understanding the unique needs of Indigenous people in Saskatchewan to further a respectful partnership with and among Indigenous community members is aligned with, and informed by, the United Nations Declaration on the Rights of Indigenous Peoples (UNDRIP)^26^ and the TRC Calls to Action.^27^ Obtaining a greater understanding of the unique characteristics, strengths, needs, and challenges impacting Indigenous people is important as we collectively work to decrease health inequity gaps and decolonize health care practice to better meet the needs of Indigenous people living in Saskatchewan. Future research should consider the unique needs of rural, remote, and Indigenous people, building on known facilitators and barriers to accessing healthcare for low back pain, to develop models of care that can specifically prioritize community-directed and culture-based strengths. Improving our understanding of perceived access barriers across the Saskatchewan population will assist in developing more effective care models that address the gaps between evidence-based guidelines and healthcare practice patterns and inform practices and policies more broadly for other geographical regions with relatively high proportions of rural and remote dwellers and Indigenous peoples.

## Appendix 1

### Chronic Back Pain Telephone Survey (March 2022)

**INTRODUCTION**

INT01.

Hello, my name is ***(FIRST NAME ONLY)*** and I am a student calling from the University of Saskatchewan. We are conducting a short 10-minute telephone survey to explore the perceived health care access barriers and facilitators among people with chronic back pain in Saskatchewan. The purpose is to learn about some of the strengths and weakness to accessing health care in Saskatchewan.

INTR02.

Is there anyone in your household who is 18 years of age or older and who is experiencing lower back pain that has lasted at least 3 months?

1. Yes, speaking  ***CONTINUE***
2. Yes, I’ll get him/her ***REPEAT INTRODUCTION AND CONTINUE***
3. Not available  ***ARRANGE CALLBACK → HIT “ESC” ON YOUR KEYBOARD***
*** REQUEST RESPONDENT FIRST NAME AND ARRANGE CALLBACK***
4. Refused to Transfer

INT03.

I would like to invite you to participate in this short survey. The name of the project is: *Living with Chronic Back Pain: A patient-led investigation of health care access challenges for back pain across the rural-urban continuum in Saskatchewan*. Participation is voluntary, and you can stop the survey at any time at which point your data will be deleted and destroyed. You can skip any questions you don’t want to answer. This call will be recorded for quality control purposes. Your responses will be combined anonymously with others once the call ends. None of the answers that you provide will be linked back to you personally. There are no known risks or personal benefits to participating in this survey. Data will be kept and stored for a minimum of 5 years after results have been published on password-protected university managed systems (ie, OneDrive and DataStore) accessible by the research team. This research project has been approved on ethical grounds by the University of Saskatchewan Behavioural Research Ethics Board. Any questions regarding your rights as a participant may be addressed to that committee through the Research Ethics Office at ethics.office@usask.ca or 1-888-966-2975. If you have any questions or concerns about the survey itself, you may contact the lead researcher Dr. Brenna Bath at brenna.bath@usask.ca or 306-966-6573.

Are you willing to participate?

1. Yes  ***CONTINUE***
2. No  ***THANK AND END INTERVIEW***
3. Later/Not right now ***ARRANGE CALLBACK*** → ***HIT “ESC” ON YOUR KEYBOARD***
*** REQUEST RESPONDENT FIRST NAME AND ARRANGE CALLBACK***

SCREEN1.

To confirm, you experience lower back pain? That is, pain localized between the bottom of the rib cage and the bottom of the buttocks, that may or may not include leg pain?

1. Yes
2. No  ***THANK AND END INTERVIEW***
3. (Refused) ***THANK AND END INTERVIEW***

SCREEN2.

How long have you had problems with your low back? Was it. . .?

***(READ LIST)***

1. Less than 3 months ***THANK AND END INTERVIEW***
2. 3 to less than 6 months
3. 6 months to less than a year
4. 1 year to less than 5 years
5. 5 years to less than 10 years
6. 10 years or more
7. (Refused)  ***THANK AND END INTERVIEW***

POSTAL.

Can I please have your postal code?

***IF RESPONDENT IS RELUCTANT, YOU CAN ASSURE THEM THAT THEIR POSTAL CODE WILL BE USED FOR STATISTICAL PURPOSES ONLY (TO UNDERSTAND DIFFERENCES BY REGION/GEOGRAPHY) AND WILL NOT BE USED TO IDENTIFY THEM IN ANY WAY.***

***ENSURE RESPONDENT PROVIDES POSTAL CODE IN PROPER FORMAT (EXAMPLE: S7N 1G4).***

***ENTER RESPONDENTS POSTAL CODE CHARACTERS*:**

1. (RECORD POSTAL CODE)

INSTR1.

We’re now going to read you a list of health care providers. Please let us know if you have ever accessed care from them for your back pain. If you haven’t, we’ll ask you to indicate why. Possible reasons may include cost, wait time, travel time, or location, you simply weren’t interested in accessing service from that provider, or you can provide us with another reason.

***(Interviewer note: is the respondent indicates they have not seen the health care provider but does not want to provide a reason or does not know the reason, please select “No, other” and type “refused” or “don’t know” respectively. Note the questions Q1A to Q1I will be asked in a random order.)***

Q1A. Your family physician

1. Yes
2. No, cost
3. No, wait time
4. No, travel time/location
5. No, not interested
6. No, other *(SPECIFY)*
7. (Don’t Know)
8. (Refused)

Q1B. A physiotherapist

1. Yes
2. No, cost
3. No, wait time
4. No, travel time/location
5. No, not interested
6. No, other *(SPECIFY)*
7. (Don’t Know)
8. (Refused)

Q1C. A chiropractor

1. Yes
2. No, cost
3. No, wait time
4. No, travel time/location
5. No, not interested
6. No, other *(SPECIFY)*
7. (Don’t Know)
8. (Refused)

Q1D. A massage therapist

1. Yes
2. No, cost
3. No, wait time
4. No, travel time/location
5. No, not interested
6. No, other *(SPECIFY)*
7. (Don’t Know)
8. (Refused)

Q1E. An acupuncture provider

1. Yes
2. No, cost
3. No, wait time
4. No, travel time/location
5. No, not interested
6. No, other *(SPECIFY)*
7. (Don’t Know)
8. (Refused)

Q1F. An Indigenous or traditional healer

1. Yes
2. No, cost
3. No, wait time
4. No, travel time/location
5. No, not interested
6. No, other *(SPECIFY)*
7. (Don’t Know)
8. (Refused)

Q1G. A surgeon/specialist

1. Yes
2. No, cost
3. No, wait time
4. No, travel time/location
5. No, not interested
6. No, other *(SPECIFY)*
7. (Don’t Know)
8. (Refused)

Q1H. An exercise therapist

1. Yes
2. No, cost
3. No, wait time
4. No, travel time/location
5. No, not interested
6. No, other *(SPECIFY)*
7. (Don’t Know)
8. (Refused)

Q1I. Community supports, services, or facilities

1. Yes
2. No, cost
3. No, wait time
4. No, travel time/location
5. No, not interested
6. No, other *(SPECIFY)*
7. (Don’t Know)
8. (Refused)

Q1J. Somewhere else?

***(Interview note: If respondent discusses barriers in their response, please ensure that information is recorded)***

1. (ENTER RESPONSE VERBATIM)
2. (Don’t Know)
3. (Refused)

Q2A. Have you taken medication to treat your back pain?

1. Yes
2. No
3. (Don’t Know)
4. (Refused)

Q2B. Have you used traditional healing methods to treat your back pain?

1. Yes, what type? (SPECIFY)
2. No
3. (Don’t Know)
4. (Refused)

INSTR3.

I’m now going to read you a list of possible factors that may or may not make it difficult for you to get the help you need for your back pain. Please let me know if the factor is not at all a barrier for you, a minor barrier, a moderate barrier, or a serious barrier to getting the help you need.

Travel to access health services

1. Not at all a barrier
2. A minor barrier
3. A moderate barrier
4. A serious barrier
5. (Don’t Know)
6. (Refused)

Q3B. Wait times

1. Not at all a barrier
2. A minor barrier
3. A moderate barrier
4. A serious barrier
5. (Don’t Know)
6. (Refused)

Q3C. Costs

1. Not at all a barrier
2. A minor barrier
3. A moderate barrier
4. A serious barrier
5. (Don’t Know)
6. (Refused)

Q3D. Cultural sensitivity

1. Not at all a barrier
2. A minor barrier
3. A moderate barrier
4. A serious barrier
5. (Not Applicable)
6. (Don’t Know)
7. (Refused)

Q3E. Comfort interacting with health care professionals

1. Not at all a barrier
2. A minor barrier
3. A moderate barrier
4. A serious barrier
5. (Don’t Know)
6. (Refused)

Q3F. Personal responsibilities for providing care to others

1. Not at all a barrier
2. A minor barrier
3. A moderate barrier
4. A serious barrier
5. (Don’t Know)
6. (Refused)

Q3G. Not knowing where to go or what will help (ie, feeling lost in the health care system)

1. Not at all a barrier
2. A minor barrier
3. A moderate barrier
4. A serious barrier
5. (Don’t Know)
6. (Refused)

Q4. Are there any other barriers you encounter that make it difficult for you to access care for your back pain?

1. YES (RECORD RESPONSE VERBATIM)
2. No
3. (Refused)

INSTR5.

I’m now going to read you a list of possible factors that may or may not make it easier for you to get the care you need for your back pain. Please let me know if the factor is not at all a help for you, a minor help, a moderate help, or a major help to getting the care you need for your back pain.

Q5A. Publicly funded health care

1. Not at all a help
2. A minor help
3. A moderate help
4. A major help
5. (Don’t Know)
6. (Refused)

Q5B. Health care nearby in your community.

1. Not at all a help
2. A minor help
3. A moderate help
4. A major help
5. (Don’t Know)
6. (Refused)

Q5C. Quick access to health care providers (ie, no or short wait time)

1. Not at all a help
2. A minor help
3. A moderate help
4. A major help
5. (Don’t Know)
6. (Refused)

Q5D. Health care provider who helps you get care (ie, refers you on for care or follow up)

1. Not at all a help
2. A minor help
3. A moderate help
4. A major help
5. (Don’t Know)
6. (Refused)

Q5E. Having additional health care insurance.

1. Not at all a help
2. A minor help
3. A moderate help
4. A major help
5. (Don’t Know)
6. (Refused)

Q5F. Telehealth/virtual care

1. Not at all a help
2. A minor help
3. A moderate help
4. A major help
5. (Don’t Know)
6. (Refused)

Are there any other factors that have made it easier for you to access care for your back pain?

7. YES (RECORD RESPONSE VERBATIM)
8. No
9. (Refused)

Q6. Over the past year or 2, healthcare practices have changed due to COVID-19. Would you say these changes have impacted you positively, negatively, or had no impact on your ability to access the care you need for your back pain?

1. Positively
2. No impact
3. Negatively
4. (Not Applicable—did not try to access services during this time)
5. (Don’t Know)
6. (Refused)

Q6A-(If positive or negative) can you tell me WHY or HOW COVID-19 has impacted your ability to access the care you need? (RECORD RESPONSE VERBATIM)

D1. Now to ensure that we are talking to a representative cross section of Saskatchewan residents, we need to get a little information about your background.

In what year were you born?

1. (ENTER YEAR OF BIRTH)
2. (Refused)

D2. With what gender do you identify?

1. Man
2. Woman
3. I prefer to self-describe as: _______
4. Prefer not to answer

D3. What is your height in feet and inches?

1. (RECORD RESPONSE)
2. (Refused)

D4. What is your weight in pounds?

1. (RECORD RESPONSE)
2. (Refused)

D5. Do you self-identify as Indigenous (First Nation, Métis, or Inuit)?

1. Yes
2. No  ***SKIP TO Q6***
3. (Refused) ***SKIP TO Q6***

D5A. Do you live on reserve land?

1. Yes
2. No
3. (Refused)

D5B. Are you a traditional Knowledge Keeper or Elder?

1. Yes
2. No
3. (Refused)

D6. What is the highest level of education that you have completed?

1. Did not complete Grade 12
2. High School
3. Trade/Technical School/College
4. Some University
5. Bachelor’s degree
6. Graduate Degree
7. (Refused)

D7. Considering your main form of work, are you currently working full time, part time, a student, retired, caring for a child or family member, unemployed, disabled, engaged in cultural or traditional work, or something else?

1. Work full-time
2. Work part-time
3. Caring for a child or family member **SKIP TO D8**
4. Unemployed  **SKIP TO D8**
5. Disabled, unable to work  **SKIP TO D8**
6. Student  **SKIP TO D8**
7. Retired  **SKIP TO D8**
8. Cultural/traditional work (EXPLAIN) **SKIP TO D8**
9. Other (EXPLAIN)  **SKIP TO D8**
10. (Refused)  **SKIP TO D8**

D7A. What is your occupation?

1. (RECORD OCCUPATION) **SKIP TO D9**
2. (Refused)  **SKIP TO D9**

D8. Are you not working because of your low back problem?

1. Yes
2. No
3. (Refused)

D9. Do you live with someone who can support you in dealing with your back pain?

1. Yes (record relationship)
2. No
3. (Refused)

D10. Do you have extra insurance coverage for health care in addition to Saskatchewan Health coverage (ie, doctors, prescriptions and in hospital care)? If yes, what type is it?

***(READ LIST IF NECESSARY)***

1. Group Health insurance (ie, coverage through work health plan, Blue Cross, other)
2. SGI funding
3. Workers Compensation Board funding
4. Short or long term disability funding
5. Other funding
6. No additional coverage
7. (Don’t Know)
8. (Refused)

D11. Could you please tell me your total annual household income from all sources in 2021. I can read you a list and just let me know where you fall.

***IF ASKED, ALL SOURCES INCLUDE EMPLOYMENT INCOME (WAGES OR SALARY), SAVINGS, PENSIONS, RENT, ETC.***

1. Less than $15 000
2. $15 000 to less than $30 000
3. $30 000 to less than $60 000
4. $60 000 to less than $100 000
5. $100 000 or more
6. (Don’t Know)
7. (Refused)

E1. The researchers have another part of the study where they are doing one-on-one interviews for around 30 minutes about experiences with back pain and accessing care. Honoraria will be provided. The information you provided to me today will not be linked to your interview responses. The researchers are hoping to recruit more Indigenous participants and/or people from remote communities. If you agree, I will collect your name and phone number and pass it on to the research team who may contact you at a later date. Would you be interested in taking part in a follow-up one-on-one interview?

1. Yes
2. No

(**If Yes, Record NAME, PHONE NUMBER**)

END.

Those are all the questions that I have! On behalf of the University of Saskatchewan, thank you for your time. Your responses are greatly appreciated! Have a great day/evening!
